# Degrees of Multidisciplinarity Underpinning Care Planning for Patients with Cancer in Weekly Multidisciplinary Team Meetings: Conversation Analysis

**DOI:** 10.2147/JMDH.S270394

**Published:** 2021-02-18

**Authors:** Tayana Soukup, Ged Murtagh, Benjamin W Lamb, James S A Green, Nick Sevdalis

**Affiliations:** 1 Centre for Implementation Science, King’s College London, London, UK; 2 Imperial College London, London, UK; 3 Department of Urology, Cambridge University Hospitals NHS Foundation Trust, Cambridge, UK; 4 Faculty of Health, Education, Medicine and Social Care, Anglia Ruskin University, Cambridge, United Kingdom; 5 Whipps Cross University Hospital, Barts Health NHS Trust, London, UK

**Keywords:** cancer multidisciplinary team meetings, cancer care, decision-making, fidelity

## Abstract

**Purpose:**

Despite an increase in research on multidisciplinary team (MDT) meetings, the implementation of MDT-driven decision-making, ie, its fidelity, remains unstudied. We report fidelity using an observational protocol measuring degree to which MDTs in their weekly meetings in the UK adhere to 1) the stages of group decision-making as per the ‘Orientation-Discussion-Decision-Implementation’ framework, and 2) cancer guidelines on the composition and characteristics of their weekly meetings produced by the UK’s Department of Health, UK’s National Cancer Action Team, Cancer Research UK, World Health Organization, and The Expert Advisory Group on Cancer to the Chief Medical Officers of England and Wales.

**Patients and Methods:**

This is a prospective cross-sectional observational study of MDT meetings in the UK. Breast, colorectal, and gynecological cancer MDTs across three hospitals in the UK were video recorded over 12 weekly meetings, respectively, encompassing 822 case-reviews. A cross-section of 24 case-reviews was analysed with the main outcomes being adherence to the ‘Orientation-Discussion-Decision-Implementation’ framework, and the cancer guidelines.

**Results:**

Eight percent of case-reviews in the MDT meetings involved all five core disciplines including surgeons, oncologists, radiologists, histopathologists, and specialist cancer nurses, and 38% included four. The majority of case-reviews (54%) were between two (25%) or three (29%) disciplines only. Surgeons (83%) and oncologists (8%) most consistently engaged in all stages of decision-making. While all patients put forward for MDT meeting were actually reviewed, 4% of them either bypassed the orientation (case presentation), and 8% did not articulate the final decision to the entire team.

**Conclusion:**

We found that, despite being a set policy, cancer case-reviews in MDT meetings are not entirely MDT-driven, with more than half of the case-reviews not adhering to the cancer guidelines, and just over 10% not adhering to the group decision-making framework. The findings are in line with the UK recommendation on streamlining MDT meetings and could help decide how to re-organise the meetings to be most efficient. Implications are discussed in relation to quality and safety of care.

## Introduction

In the United Kingdom (UK), as well as in many other countries worldwide, multidisciplinary teams (MDTs) are integral to care planning for people with cancer.^1–5^ Their regular weekly meetings (also known as multidisciplinary tumor boards or conferences) are embedded into the care pathways and structures of the UK National Health Services (NHS). In the UK, the core disciplines in attendance include oncologists, surgeons, radiologists, pathologists, and cancer nurse specialists (CNS) who convene to review patients’ clinical details and formulate a care plan.^1–4^ These five clinical disciplines are required to attend and contribute to care planning for all patients on the meeting agenda.^1–4^ Such MDT-driven decision-making stemmed from the Calman-Hine report^4^ in 1995 highlighting variation in the delivery of cancer care in the UK that persists today.^3^,^6^ MDT meetings are now a mandatory component of the cancer care pathway in the UK, driving treatment planning for people with cancer.^1–4^,^6^

Guidelines for cancer MDTs, including those by the UK’s Department of Health,^1^ UK’s National Cancer Action Team,^2^ Cancer Research UK,^3^ and the Expert Advisory Group on Cancer to the Chief Medical Officers of England and Wales^4^ set standards on team composition, characteristics, and functionality of MDT meetings. The guideline recommendations are that cancer care should be driven by a multidisciplinary team who should meet regularly, for example on a weekly or fortnightly basis, and comprise a range of professionals necessary for care planning, including surgeons, radiologists, histopathologists, oncologists, and in the UK also cancer nurse specialists. In the UK, the minimum number of disciplines needed to convene a multidisciplinary team meeting are six, ie, surgeons, radiologists, histopathologists, oncologists, cancer nurse specialists, and a team coordinator who has an administrative role. All five core clinical disciplines are expected to contribute to care planning for patients on the meeting agenda (their attendance is registered and monitored).^1–4^ Unlike in some countries, in the UK, the allied health professionals, such as psychologists and speech therapists are not considered core cancer MDT members, although they do attend some cancer MDT meetings, such as for example, head and neck.

However, the degree to which the MDT approach to care planning in their weekly meetings is implemented as intended, ie, its fidelity,^7^ is understudied.^8^ Currently, there is no developed methodology for capturing the fidelity of MDT decision-making in these meetings. Generally, there are a few examples of established procedures for monitoring fidelity of changes/innovations as delivered in actual clinical practice,^9^,^10^ and none in the context of MDT meetings. Yet, it is understood that such innovations can sometimes fail when implemented on a wide scale.^10^

**Figure 1:**
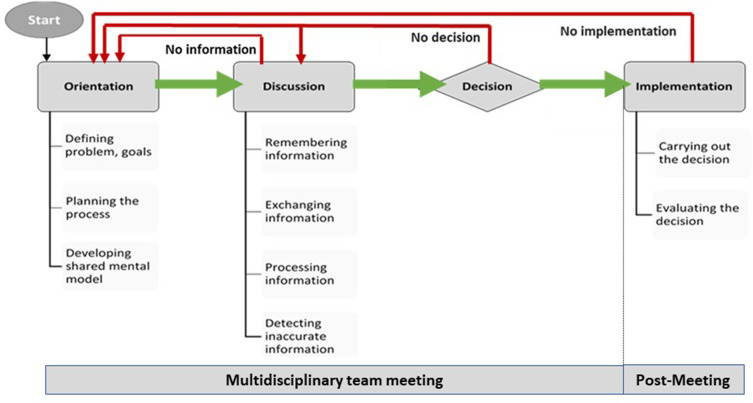
Orientation-Discussion-Decision-Implementation (ODDI) Framework of Group Decision-Making. **Notes:** Adapted with permission from Forsyth DR. *Group Dynamics*. US: Jon-David Hague; 2014.^43^ Reprinted with permission from Soukup T. Socio-cognitive factors that affect decision-making in cancer multidisciplinary team meetings [PhD Thesis; Clinical Medicine Research]. London, UK: Imperial College London; 2017.^37^

So far, the research points to poor implementation of MDT decision-making at the point of their weekly meetings.^8^,^11–20^ Asymmetries in participation and suboptimal sharing of information between team members in the meetings are of particular concern since complete patient profile (patients’ comorbidities, psychosocial aspects, and views on treatment options) and input by all core disciplines (ie, CNSs, surgeons, radiologists, histopathologists, and oncologists) are needed for the MDT to reach a recommendation and subsequently implement it.^14–17^,^21^,^22^ With an estimated cost of MDT meetings at £100 million a year,^23^ inefficient or insufficient communication and decision-making at the point of the meeting may inadvertently add to the existing pressures, such as frequency/duration of meetings (esp. for large teaching hospitals),^24^ workload,^25^ financial pressures,^26^ and complexity of care pathway for patients.^27^ Red arrows in Figure 1 graphically represent this argument where it can be seen that if the MDT 1) does not have enough information about the patient at the point of the meeting, 2) it will not be able to reach a decision, and 3) it will subsequently not be able to implement that decision with the patient, ie, the patient case will be put forward to another MDT meeting adding to the team workload, complexity of the care pathway for the patient, and the associated financial pressures. As cancer MDTs are trying to maximize productivity in the face of increasing workloads and pressures, safety concerns have been raised in the UK in the context of MDT meetings with one-member reporting “Sometimes we discuss up to 70 patients. This is after a whole day of clinics and we don’t finish until gone 19.00. Would you want to be number 70?”^3^

Team science offers frameworks and methods for understanding interaction and decision-making and how best to employ these to improve team effectiveness and efficiency in work meetings. Other reliability industries, such as aviation, have successfully employed them.^28–30^ One example is the framework of group decision-making, namely, the “Orientation-Discussion-Decision-Implementation” (ODDI; green arrows in Figure 1), which offers a pragmatic approach to understanding, evaluating, and improving MDT decision-making. It stipulates that team decision-making should progress in a logical manner: 1) identifying and defining the problem/question (orientation); 2) sharing and evaluating information (discussion); 3) combining the input from members into a decision (decision); and 4) implementing and evaluating the decision by feeding the reasons for (non)-implementation back to the team, as a vehicle to improving team processes.^31–34^ Adherence to the stages of the ODDI leads to more effective performance, ie, better decision-making quality, improved variability, and high level of task efficiency. For example, the more time a group spends on building an accurate understanding of the problem/question (orientation), the better the proceeding discussion, which then directly predicts the team’s ability to reach and implement their decision.^31–35^

Whereas the ODDI appears to offer a logical and suitable approach, no study to-date has applied it to the MDT decision processes in cancer care at the point of their weekly meetings. The extent and the quality of adherence to the individual stages in MDT meetings is therefore unknown. Building of such an evidence-base would be uniquely useful: it would allow assessment of MDT decision-making fidelity in the context of their weekly meetings in a structured manner according to the individual stages, while “diagnosing” what element(s) of this process require attention, and facilitating improvement through team feedback.^19^ In turn, such knowledge would contribute to accurate interpretation of team outcomes, enhancing understanding of how MDT-driven care planning works in practice, and aid identification of MDT needs and aspects of their delivery requiring improvement.

## Methods

To ensure reporting rigor, we followed the STROBE checklist.^36^

### Study Aim

To develop a novel method for assessing MDT decision-making fidelity at the point of their weekly meetings by addressing the following:

1. To what degree do MDTs adhere to stages of group decision-making, as per the ODDI?^29–32^
2. To what degree is MDT input into case-reviews multidisciplinary, as per the guidelines?^1–5^

### Study Design and Setting

This was a prospective cross-sectional observational study. It took place across three hospitals in the Greater London and Derbyshire areas in the UK between September 2015 and July 2016. These were teaching hospitals, which deliver NHS services, train the future workforce, and advance patient treatment through ground-breaking research. Three cancer MDTs took part: breast, colorectal, and gynecological. Their weekly MDT meetings were video recorded for 12 consecutive weeks each. The first two meetings from each MDT were excluded from the analysis to allow teams to get used to the camera, minimizing any Hawthorne effect.

### Participants and Cases

Participants were 44 members of the three participating cancer MDTs (breast MDT=15; colorectal MDT=15; gynecological MDT=14). The MDTs had the same composition: surgeons (n=12), oncologists (n=6), CNSs (n=12), radiologists (n=6), histopathologists (n=5), and coordinators (n=3). Members were at senior level during the study period with on average 9 years of experience (min=2, max=22). Detailed breakdown of team composition is in Table 1.^37^,^38^

**Table 1:** Team Composition and Meeting Characteristics Across the Participating Cancer Multidisciplinary Teams

	Breast MDT			Colorectal MDT			Gynecological MDT		
	N	M	Min, Max	N	M	Min, Max	N	M	Min, Max
**Team composition**									
Surgeons	4	4	4, 4	4	4	4, 4	4	2	2, 4
Oncologists	2	2	2, 2	2	1	1, 1	2	1	1, 1
Radiologists	2	1	1, 2	2	1	1, 1	3	1	1, 1
Histopathologists	1	1	1, 1	1	1	1, 1	2	1	1, 1
Specialist cancer nurses	5	2	2, 4	5	3	2, 4	2	1	2, 4
Team coordinator	1	1	1	1	1	1	1	1	1, 1
Total	15	11	–	15	11	–	14	7	–
**Meeting characteristics**									
Average number of cases discussed per meeting	26			20			43		
Average time per patient (HH:MM:SS)	00:02:25			00:03:20			00:02:30		
Average meeting duration (HH:MM:SS)	01:06:00			01:00:00			02:52:00		
**Study characteristics**									
Number of hours recorded (HH:MM:SS)	09:57:00			13:40:00			31:30:00		
Number of cases discussed	241			185			396		
Number of meetings observed	10			10			10		

**Notes:** Adapted with permission from Soukup T. *Socio-Cognitive Factors That Affect Decision-Making in Cancer Multidisciplinary Team Meetings [Phd Thesis; Clinical Medicine Research]*. London, UK: Imperial College London; 2017.^38^ and Soukup T, Murtagh G, Bali A, et al. Gaps and overlaps in healthcare team communication: analysis of speech patterns in cancer multidisciplinary meetings. *Small Group Res*. 2020:1–31.^37^

**Abbreviations:** N, total number of MDT members; M, average number of MDT members in attendance; Min, Max, minimum and maximum number of MDT members in attendance.

All cases put forward for MDT discussion were filmed. The final dataset comprised 30 meetings, 822 case-reviews, and 55 hours of meeting footage. Of those, and in light of the complexity of the analyses, a selection of 24 malignant case-reviews is presented here (eight patients per MDT) totaling 72 minutes of footage. The selected case-reviews were transcribed by an independent conversation analyst using Jefferson notation^39^,^40^ with all names changed to preserve confidentiality. The selection criteria for the 24 case discussions was as follows:

1. audio quality and clarity for transcription using complex Jefferson notation,^39^,^40^ since analysis of multiparty conversations can be difficult due to the problem of differentiating inputs from overlapping speakers (also the criteria in Dew,^41^ study on MDT meetings);
2. feasibility, since a) the transcription using Jefferson notation is complex and resource intense (especially for multiple speakers), and b) the method of analyzing the fidelity in MDT meetings presented in this study is novel utilizing qualitative data extracts common in Conversation Analysis and quantitative frequency counts common in language sciences, hence the subset was limited to 24 cases;
3. malignancy, since benign cases are also discussed at some MDT meetings, and due to the nature and duration of these discussions, it was important to distinguish between malignant and non-malignant cases (only malignant cases were included);
4. duration of the case discussion, since this can vary from case to case, and is important to consider when using frequencies (hence, the selected cases were similar duration, 00:02:25 to 00:03:25);
5. 1st and 2nd half of the meeting ensuring equal distribution of case discussion across the meeting duration (four case discussions were selected from each half across teams);
6. saturation on the basis of the case discussions that have met the above criteria.

For the selected cases, the question for the MDT was broadly defined as “for MDT review”. Cases with a more specific question, such as those that require radiologist’s, histopathologist’s, or oncologist’s input specifically, were not included in this analysis.

MDTs and their members were recruited into the study through the National Institute for Health Research (NIHR), Clinical Research Network (CRN), and the NIHR Central Portfolio Management System (CPMS) who adopted the study and opened it for recruitment to all cancer MDT members in England. This allowed interested MDT members to get in touch directly with the researcher, while also enabling the local NIHR CRN/CPMS teams to directly introduce the study and recruit MDTs from their Trusts. The recruitment was further enhanced by opportunistic sampling through the existing networks within the academic Department of Surgery and Cancer situated within a large teaching hospital that hosted the study.

The study was granted ethical and regulatory approval by the North West London Research Ethics Committee (JRCO REF. 157441), and also locally by the R&D departments of the participating NHS Trusts. Informed consent was sought from all participants. The study was adopted by the National Institute for Health Research Clinical Research Network Portfolio.

### Method of Analysis

The recommended “gold standard” strategy for assessing fidelity of delivery involves objectively verifying delivery by comparing the content of recorded intervention sessions to a pre-specified criterion such as a manual, guidelines, and/or frameworks.^10^,^42^ The fidelity of MDT decision-making was assessed against two matrices.

### Q1: Adherence to the Stages of Group Decision-Making in MDT Meetings

The first matrix was the frequency and consistency of involvement in the stages of the ODDI framework (Figure 1) by the core disciplines, ie, CNSs, surgeons, oncologists, radiologists, and histopathologists. This is how we defined each ODDI stage:

Orientation is presenting (the case) and identifying, defining, and developing a clear understanding of what needs to be addressed by the team; setting and clarifying the problem/question/goal for the team and planning how to address it by identifying resources and information needed; eg, introducing a patient to the team so that everyone is aware of who is about to be discussed, identifying why they are on the agenda, and what the goal/problem/question is for the team in relation to the case (eg, a review of pathology or radiology results).

Discussion is remembering, exchanging, retrieving, seeking information and opinions, examining alternatives, strengths and weakness of different options, and detecting errors and inaccuracies.

Decision is where opinions are combined into a single group decision which is named and announced to the team.

Implementation is when the decision made by the team is applied post-MDT meeting; this stage was outside the scope of the research, as we only collected data in vivo during the MDT meetings, and did not follow up the MDT-agreed clinical actions.

### Q2: Adherence to the Cancer Guidelines in MDT Meetings

The second matrix was frequency and consistency of input into case-reviews by the core disciplines. In line with the cancer guidelines,^1–4^ in the UK, the minimum number of core disciplines formally required to be present at the meetings was six: CNSs, oncologists, radiologists, histopathologists, surgeons, and coordinators. In the UK, cancer nurse specialists (CNSs) are considered a core MDT member group required to attend MDT meetings and contribute to care planning for all patients on the meeting agenda. MDT meeting can only commence once these five clinical disciplines are present, ie, cancer nurse specialists, surgeons, oncologists, radiologists, and histopathologists. On the other hand, in the UK, the MDT coordinator has an administrative role in MDT meetings, and is in charge of recording the outcome of team discussion in the electronic records for each patient, hence they are always seated at the computer in the meeting room. They are also in charge of organizing the meetings and ensuring all paper files for patients are available to the core clinical members of the team. Equally, an MDT meeting cannot commence until the coordinator is present.

Hence, six is the minimum number of professional groups (cancer nurse specialists, surgeons, oncologists, radiologists, histopathologists, and coordinators) that must be present to constitute an MDT meeting in the UK, while five is the minimum number that must contribute (cancer nurse specialists, surgeons, oncologists, radiologists, and histopathologists) to care planning for patients on the meeting agenda. This is the guideline standard that we have assessed our cases against. In our analysis therefore, a multidisciplinary case-review would need all five clinical disciplines, ie, cancer nurse specialists, surgeons, oncologists, radiologists, and histopathologists that formally attend to engage in decision-making in the meetings. For example, if the surgeon and pathologist engaged in the decision-making process, as per the ODDI framework, during a particular case-review in the meeting, then it is concluded that the case-review was underpinned by a disciplinary-dyad (surgeon-pathologist pair). If three different disciplines engaged then it was termed a disciplinary-triad, and if there were four a tetrad. It is important to note that we considered the extent of involvement of the core disciplines at any stage of the ODDI framework. For example, a radiologist could be engaged in the discussion stage only, and their engagement would still be considered adherent to the ODDI framework.

### Data Analysis Procedure

Engagement in case reviewing in the meetings was defined as verbal contribution to case reviewing. It was determined using Jefferson transcription system^39^ commonly used in Conversation Analysis;^40^ focus of the transcription and analysis was on verbal means of communication as opposed to non-verbal. Transcription using Jefferson notation provides detailed data on the complex nature of communication and engagement in group interaction. A combination of qualitative data extracts and quantitative frequency counts were used for analysis. For quality control, the data have been discussed in multiple data sessions (N=4) with leading international communication scholars who provided their critical input and insight into the analysis presented in this study. In a confidential manner, the scholars watched MDT meeting videos and discussed the communication, while formulating points of interest in the data. This is seen as a routine piece of scholarly teamwork, and a vital part of conversation and interaction analysis.

Based on the level of engagement in case reviewing established using Jefferson notation^39^ and principles of Conversation Analysis,^40^ the decision-making formats were identified inductively using a thematic approach where the themes ie the decision-making formats emerged directly from the data. We grouped each of the 24 case transcripts according to the two matrices described in detail above, ie, adherence to the stages of group decision-making, and degree of multidisciplinarity as set by the guidelines. We then labeled each theme representing a specific decision-making format. For quality control and face validity, the final set of decision-making formats was discussed with the experts in the field of cancer MDTs.

## Results

### Q1: Adherence to the Stages of Group Decision-Making in MDT Meetings

Table 2 shows relative frequencies (%) according to a) all 24 case-reviews and b) each core clinical discipline in MDT meeting attendance. The number of core disciplines across teams varied, with MDT coordinators and pathologists showing the smallest group sizes, and surgeons and CNSs being the largest. Surgeons and oncologists consistently engaged in all stages of the ODDI framework with additional disciplines involved in the discussion only; the exception were CNSs who also engaged in orientation stage, although to a lesser extent. Table 2 also reveals that a small percentage of case-reviews (4%) bypassed the orientation stage, and went directly into discussing the patient. A higher percentage (8%) of case-reviews did not appear to name or announce the final decision at the end of a discussion.

**Table 2:** Frequency of Adherence by Core Disciplines to the Orientation-Discussion-Decision-Implementation Framework of Group Decision-Making

Core Discipline*	N	ODDI Framework of Group Decision-Making			
		Orientation (% of cases)	Discussion (% of cases)	Decision (% of cases)	All stages consistently (% of cases)
Surgeon	12	83	83	88	83
Oncologist	6	13	42	21	8
Radiologist	7	0	58	0	0
Histopathologist	4	0	71	0	0
Cancer Nurse Specialist	12	4	46	0	0
MDT Coordinator	3	0	4	0	0
Case-reviews*	24	96	100	92	88

**Notes:** *Across all three cancer teams combined (colorectal, breast, and gynecological cancer MDTs). ODDI=Orientation-Discussion-Decision-Implementation; % values rounded to nearest integer for ease of reading. Reprinted with permission from Soukup T. *Socio-Cognitive Factors That Affect Decision-Making in Cancer Multidisciplinary Team Meetings [Phd Thesis; Clinical Medicine Research]*. London, UK: Imperial College London; 2017.^38^

### Q2: Adherence to the Cancer Guidelines in MDT Meetings

#### Breast Cancer Team Meetings (Table 3A)

This team was predominantly surgeon-oncologist led where either one of them consistently engaged in all stages of team decision-making in their weekly meetings, as per the ODDI framework. During the discussion stage in the meeting, interactions occurred within tetrads with various combinations of disciplines. In contrast, the orientation and decision stages in the meeting were distributed between surgeons and oncologists only. However, 4% of case-reviews bypassed the orientation stage and went directly into discussing the patient.

**Table 3:** Multidisciplinary Interaction Formats Underpinning Case-Reviews in Breast, Colorectal, and Gynecological Cancer Multidisciplinary Team Meetings Together with the Frequency of the Formats Across 24 Case Reviews

**3A. Breast Cancer Multidisciplinary Team**																										
**Surgeon-Oncologist-Led Decision-Making** (62%)																	**Surgeon-Led Decision-Making** (38%)									
**Disciplinary-triad** (13%)			**Disciplinary-tetrad** (38%)							**Multi-disciplinary** (13%)							**Disciplinary-triad** (13%)						**Disciplinary-tetrad** (25%)			
Case 2		Orientation: S Discussion: O-P-S Decision: S-O	Case 6			Orientation: O Discussion: O-S-P-R Decision: S-O				Case 12		Orientation: S Discussion: O-S-P-R-N Decision: O-S					Case 16	Orientation: S Discussion: O-P-S Decision: S					Case 8			Orientation: S Discussion: R-P-S-N Decision: S
			Case 20			Orientation: O Discussion: O-S-P-R Decision: S																	Case 15			Orientation: S Discussion: R-P-S-N Decision: S
			Case 10			Orientation: -* Discussion: O-S-P-N Decision: S-O																				
**3B. Colorectal cancer multidisciplinary team**																										
**Surgeon-led decision-making** (100%)																										
**Disciplinary-dyad** (13%)							**Disciplinary-triad** (38%)								**Disciplinary-tetrad** (38%)						**Multi-disciplinary** (13%)					
Case 13		Orientation: S Discussion: S-R Decision: S					Case 2		Orientation: S Discussion: S-R-N Decision: S						Case 3	Orientation: S Discussion: S-P-N-C Decision: S					Case 12				Orientation: S Discussion: S-P-N-R-O Decision: S	
							Case 4		Orientation: S Discussion: S-R-P Decision: S						Case 5	Orientation: S Discussion: S-P-N-R Decision: S										
							Case 14		Orientation: N-S Discussion: S-R-N Decision: S						Case 11	Orientation: S Discussion: S-P-N-R Decision: S										
**3C. Gynaecological cancer multidisciplinary team**																										
**Surgeon-led decision-making** (87.5%)																				**Oncologist-led decision-making** (12.5%)						
**Disciplinary-dyad** (63%)					**Disciplinary-triad** (13%)								**Disciplinary-tetrad** (13%)							**Disciplinary-triad** (25%)						
Case 1	Orientation: S Discussion: S-P Decision: S				Case 45			Orientation: S Discussion: S-R-O Decision: S					Case 37	Orientation: S Discussion: S-R-O-N Decision: not named*					Case 26			Orientation: O Discussion: O-R-S Decision: O				
Case 27	Orientation: S Discussion: S-P Decision: S																									
Case 2	Orientation: S Discussion: S-P Decision: S																									
Case 4	Orientation: S Discussion: S-P Decision: not named*																									
Case 5	Orientation: S Discussion: S-P Decision: S																									

**Notes:** *Not named/announced to the team. Disciplinary-triad=interaction between three core disciplines. Disciplinary-tetrad=interaction between four core disciplines. Multidisciplinary=interaction between five or more core disciplines. One person per discipline was engaged in each case. Reprinted with permission from Soukup T. *Socio-Cognitive Factors That Affect Decision-Making in Cancer Multidisciplinary Team Meetings [Phd Thesis; Clinical Medicine Research]*. London, UK: Imperial College London; 2017.^38^

**Abbreviations:** S, surgeon; R, radiologist; P, pathologist; N, cancer nurse specialist; O, oncologist.

#### Colorectal Cancer Team Meetings (Table 3B)

This team was surgeon-led, ie, surgeons consistently engaged in all stages of team decision-making in their weekly meetings, as per the ODDI framework. During the discussion stage in the meeting, interactions occurred predominantly within triads and tetrads and included a diverse mix of core disciplines. Orientation in the meeting was mainly led by surgeons, and to a lesser extent by CNSs, however, the decision resided solely with surgeons.

#### Gynaecological Cancer Team Meetings (Table 3C)

This team was also surgeon-led, ie, surgeons consistently engaged in all stages of team decision-making in their weekly meetings. The interactions during the discussion in the meeting largely occurred within dyads, ie, surgeon–pathologist pairs, although disciplinary-triads and tetrads also occurred and tended to include radiologists, surgeons, and pathologists. Orientation and decision rested predominantly with surgeons, and to a lesser extent with oncologists. In 4% of cases the decision was not named or announced to the team (Table 3).

#### Overall Dataset (Table 4 and Box 1)

Table 4 shows that case-reviews in MDT meetings were not entirely multi-disciplinary: only 8% of overall discussions involved all five clinical disciplines, and 38% included four. The majority, ie, 54% of the interactions studied, took place between two (25%) or three (29%) disciplines only. As far as the individual teams are concerned, there was variability in the number of disciplines involved in individual case-reviews, with the gynaecological team meetings showing a predominantly dyadic interaction format that occupied 63% of the corpus. Tetrads appeared most frequently in breast and colorectal team meetings with the latter also showing a high percentage of triadic reviews. Surgeon-led discussions in the meetings were most frequent overall (75%), particularly in the colorectal and gynecological team meetings, whilst the breast team meetings showed strong oncologists’ involvement, with 63% of discussions led jointly by both disciplines.

**Table 4:** Multidisciplinary Interaction Formats Across Teams and Case-Reviews

Team	Disciplinary Engagement*	Disciplinary Interaction Format				Overall
		Dyad	Triad	Tetrad	Multi	
**Breast**	Surgeon-led	-	13%	**25%**	-	**38%**
	Oncologist-led	-	-	-	-	-
	**Surgeon-Oncologist-led**	-	13%	**38%**	13%	**63**%
	Overall	-	25%	**63%**	13%	100%
**Colorectal**	**Surgeon-led**	13%	**38**%	**38%**	13%	**100**%
	Oncologist-led	-	-	-	-	-
	Surgeon-Oncologist-led	-	-	-	-	-
	Overall	13%	**38%**	**38%**	13%	100%
**Gynaecological**	**Surgeon-led**	**63**%	13%	12.5%	-	**88**%
	Oncologist-led	-	13%	-	-	13%
	Surgeon-Oncologist-led	-	-	-	-	-
	Overall	**63%**	25%	13%	-	100%
**Overall**	**Surgeon-led**	**25**%	21%	**25%**	4%	**75%**
	Oncologist-led	-	4%	-	-	4%
	Surgeon-Oncologist-led	-	4%	13%	4%	21%
	Overall	25%	**29**%	**38**%	8%	100%

**Notes:** N=24 patients. *Disciplines that consistently engage in all stages of the orientation-discussion-decision-implementation (ODDI) framework and therefore lead/dominate a case-review. In bold are the highest percentages. Reprinted with permission from Soukup T. *Socio-Cognitive Factors That Affect Decision-Making in Cancer Multidisciplinary Team Meetings [Phd Thesis; Clinical Medicine Research]*. London, UK: Imperial College London; 2017.^38^

Further to Table 4, the thematic analysis of the case-reviews indicated eight specific interaction formats underpinning decision-making in the MDT meetings of the respective three teams. This is presented in Box 1 together with the frequency of the formats across 24 case-reviews.

**Box 1:** Interaction Formats Underpinning Decision-Making in Multidisciplinary Meetings of the Respective Three Teams (Gynecological, Breast, and Colorectal Cancer MDTs) Together with the Frequency of the Formats Across 24 Case-Reviews

**A. Single-Discipline Led Case-Reviews (67%)**
**Style A1**. Surgeon presented the patient case, discussion ensued within a disciplinary dyad, triad, tetrad or (the least common) multidisciplinary, surgeon made and stated the decision, 62%
**Style A2**. Oncologist presented the case, discussion ensued within a disciplinary tetrad, oncologist made and stated the decision, 4%
**B. Dual-discipline led case-reviews (21%)**
**Style B3**. Surgeon presented the case, discussion ensued within a disciplinary triad, oncologist and surgeon made and stated the decision, 8%
**Style B4**. Oncologist presented the case, discussion ensued within a disciplinary tetrad, surgeons and oncologist made and stated the decision, 4%
**Style B5**. Oncologist presented the case, multidisciplinary discussion ensued, surgeon makes and stated the decision, 4%
**Style B6**. Surgeon and CNS presented the case, discussion ensued within a triad, surgeon made and stated the decision, **4%**
**C. Incomplete decision-making process (13%)**
**Style C7**. No case presentation, discussion ensued within a disciplinary tetrad, surgeon and oncologist made the decision, 4%
**Style C8**. Surgeon presented the case, discussion ensued within a tetrad, decision was not stated verbally to the team, 8%

**Notes:** Adapted with permission from Soukup T. *Socio-Cognitive Factors That Affect Decision-Making in Cancer Multidisciplinary Team Meetings [Phd Thesis; Clinical Medicine Research]*. London, UK: Imperial College London; 2017.^38^

## Discussion

The study aimed to develop for the first time a novel method for assessing fidelity of MDT decision-making at the point of their weekly meetings. We did this by applying the ODDI framework to a range of MDT case-reviews in their meetings (Q1). We found that although all patients in the study were consistently reviewed by an MDT (as mandated in the UK), they did not consistently adhere to the stages of group decision-making. In the meetings, the orientation stage was bypassed in 4% of cases as the team went straight into discussing the patient, and for 8% of cases, the decision was not clearly stated upon completion of the team’s deliberation. This corroborates previous research showing that decisions are not always explicitly stated in MDT meetings,^41^ and when time pressures exist, the orientation stage can be compromised, as indicated by wider literature on group decision-making.^33^

In terms of MDT-driven care planning in their weekly meetings (Q2), contrary to MDT policy recommendations, multidisciplinary case-reviews were the least common (8%), indicating poor fidelity, ie, adherence to guidelines. The majority (54%) of case-reviews in MDT meetings took place between two or three disciplines: gynecological meetings appeared least multidisciplinary with dyads most frequent, while triads were more common in colorectal, and tetrads in breast cancer meetings. Disciplines most consistently engaged in all stages of group decision-making in the meetings were surgeons, oncologists, and CNSs with the case-reviews led either by surgeons and oncologists, or both, and to a lesser extent surgeons and CNSs. This is supported by previous studies on MDT meetings showing asymmetries in participation with surgeons and oncologists most commonly contributing to case-reviews.^11–20^ This is also an expected finding since some disciplines will not be involved in the orientation (ie, case presentation) or decision stages if they are not directly involved in the patient’s day-to-day care (eg, radiologists and histopathologists).

### Implications

It is important to consider the potential implications of the findings within the wider eco-political landscape, such as the increasing financial pressures on healthcare,^4^,^26^ rise in cancer incidence,^4^,^25^ and staff shortages^44^ with MDT meetings adding to these pressures.^23^,^24^,^27^ Having 54% of case-reviews taking place between only two or three disciplines in the meetings means that the implementation of current best practice (nationally mandated in the UK and many other countries) is not consistent with the spirit of the policy. Technically, the UK MDT policy implementation, as revealed in the present study, is implemented with low fidelity. Low fidelity, as in any complex intervention implementation, means that the presumed benefits of the policy may not be achieved. In the case of cancer MDTs, the policy aim was to ensure that collective team expertise and peer-review would be available for all cancer patients at the point of decision-making about their care. But, as implemented, the current study suggests that the “team” at the point of their weekly meeting consists of a few disciplines conversing with one another, rather than the intended wider group (in some MDTs at least). Such asymmetries in participating have been shown previously, indicating that a true interdisciplinary approach is not reached and this, in turn, might reduce chances of attaining a true patient-centred care plan.^8^,–^11–20^

A potentially more efficient way of organizing the cases for MDT meetings could be streamlining according to clinical complexity.^45–47^ Indeed, recent UK guidance for cancer MDTs recommends that hospitals implement such streamlining to their meetings to reduce the number of patients discussed by concentrating on those who would benefit most from full MDT review.^46^,^47^ For example, cases could be organized so that those of high complexity are reviewed by the full MDT, while those falling neatly into predetermined guidelines, are reviewed in a smaller meeting (eg, between surgeons and oncologists, as observed in our study).^46^,^47^ As current policies shift to address pressures on resources, this would mean that the MDT principles need not be abandoned, but their practical implementation requires improvement.

Our data also showed that a small number of case-reviews in MDT meetings either bypassed the orientation stage (ie, case presentation and problem/question identification), or did not explicitly articulate the recommendation. This means that members with no immediate access to patient notes or little knowledge of the patient are left “in the dark” to meaningfully contribute to discussion. Similarly, if the recommendation is not clearly announced in the meeting, the decision that is intended for the patient may not be clearly and accurately recorded by those documenting the outcomes (eg, coordinator), and/or understood by those in contact with the patient. MDT policy^2^ in the UK suggests that at the very least the patient’s name or hospital number should be announced to the team, and that a clear recommendation should be produced from an MDT review. The MDT decision can only be as good as the information it is based on.^2^ Hence sufficient information is paramount to safe and effective communication and decision-making, particularly since the ability to reach and implement the decision in the meeting is facilitated by a clearly formulated problem/question for the team.^31–33^

## Limitations

Our findings should be interpreted against certain limitations. First is the Hawthorne effect, a natural limitation to all observational studies, which we minimized by: 1) adopting a long-term approach to filming (3 months per team), 2) excluding the first two meetings per team from analysis to induce habituation, and 3) ensuring that filming was done discretely using a small camera (GoPro) positioned so it blends in with the meeting equipment. Second is silence in the meetings, which can be difficult to interpret since the current study employed observational methodology that relies on verbal behaviors.^11^ Hence future studies should look at non-verbal engagement and means of communication between team members (eg, gestures, eye contact) using Conversation Analysis,^40^ since it is methodologically suited to examine this in detail. Third is a limited number of case-reviews presented in the study (N=24). This is because we piloted for feasibility a novel method of analyzing fidelity of decision-making in these teams, paving the way for future studies focused on cancer MDT meetings (and other chronic conditions that use MDT meetings for care planning, such as mental health, for example). Such studies could apply this now tested method to a dataset larger than the current study to help build knowledge-base and generalizability. A final limitation is the generalizability of the findings outside the settings in which the study was carried out, ie, the UK’s NHS, where MDT-driven care is over 20 years old, hence an embedded policy, with specific guidelines for how MDT meetings are to run, be attended and followed up. The laborious nature of the data analysis meant we could only assess a small sample of teams and case-reviews. In addition, generalizability can also be affected by the significant time lag between when the study took place and reporting of the results (study took place between 2015 and 2016). However, there were no significant systemic changes in MDTs in the UK in the intervening time that would result in limited reproducibility.

Our study also has strengths. As real-time observations of MDT meetings, we captured the flow of behavior in its own setting, achieving greater ecological validity. We also demonstrate a novel method for assessing fidelity of MDT decision-making in their weekly meetings that can bring new insights for team improvement, and better understanding of clinical teams making high-stakes decisions for patient care in general.

### Further Research

While the implementation stage of group decision-making was beyond the scope of the current study, it is an important element further research could profitably investigate. It allows the applied MDT decision to be evaluated against the consequences of the choices made in the MDT meeting. As such, it has a significant learning and development value that could enable MDTs to improve their decision-making. MDTs should record and monitor accuracy of their prognostic judgments to improve care.^48^,^49^ Hence, reasons for non-implementation (1–16% of cases) or change (2–52% cases) of MDTs’ recommendations^12^,^15^ are valuable learning points. While previous research with MDT members has revealed that better case preparation and streamlining could help improve decision implementation,^15^ a clear understanding by a team as to why their recommendation made at the point of the MDT meeting has not been implemented may help them self-correct. This could prompt evaluation and training for improved performance, efficiency, and quality of care. As such, the ODDI framework could be used to facilitate “team audit and feedback” by diagnosing where MDTs could be doing better, and tailoring interventions to facilitate improvement.

## Conclusion

Despite being a set policy, cancer case-reviews at the point of weekly MDT meetings are not entirely MDT-driven. Our study provides a feasible approach to assessing fidelity of decision-making that could help cancer MDTs re-organize their processes to be most efficient given the resources. In line with current guidance, streamlining patient selection for MDT review could help increase the fidelity of the MDT approach in cancer care.

## Data Sharing Statement

The anonymized transcripts supporting this study are available in Zenodo, a secure data repository (https://zenodo.org/record/582283#.Xm_KlKj7Q2w), under the Creative Commons Attribution Non-Commercial Non-Derivative 4.0 license.^42^ Researchers are free to reuse and redistribute the data set on the condition that they attribute it, that they do not use it for commercial purposes, and that they do not alter it. For any reuse or redistribution, researchers must make clear to others the license terms of this work and reference the dataset accordingly.
